# Clinical-MRI radiomics enables the prediction of preoperative cerebral spinal fluid dissemination in children with medulloblastoma

**DOI:** 10.1186/s12957-021-02239-w

**Published:** 2021-04-22

**Authors:** Hui Zheng, Jinning Li, Huanhuan Liu, Chenqing Wu, Ting Gui, Ming Liu, Yuzhen Zhang, Shaofeng Duan, Yuhua Li, Dengbin Wang

**Affiliations:** 1 Department of Radiology, Xinhua Hospital affiliated to Shanghai Jiao Tong University School of Medicine, Shanghai, China; 2 GE Healthcare, Pudong New Town, No.1, Huatuo Road, Shanghai, 210000 China

**Keywords:** Children, Cerebral spinal fluid dissemination, Magnetic resonance imaging, Medulloblastoma, Radiomics

## Abstract

**Background:**

Medulloblastoma (MB) is the most common pediatric embryonal tumor. Accurate identification of cerebral spinal fluid (CSF) dissemination is important in prognosis prediction. Both MRI of the central nervous system (CNS) and CSF cytology will appear false positive and negative. Our objective was to investigate the added value of preoperative-enhanced T1-weighted image-based radiomic features to clinical characteristics in predicting preoperative CSF dissemination for children with MB.

**Materials and methods:**

This retrospective study included 84 children with histopathologically confirmed MB between November 2006 and November 2018 (training cohort, *n*=60; internal validation cohort, *n*=24). A set of cases between December 2018 and February 2020 were used for external validation (*n*=40). The children with normal head and spine magnetic resonance images (MRI) and no subsequent dissemination in 1 year were diagnosed as non-CSF dissemination. The CSF dissemination was manifested as intracranial or intraspinal nodular-enhanced lesions. Clinical features were collected, and conventional MRI features of preoperative head MRI examinations were evaluated. A total of 385 radiomic features were extracted from preoperative-enhanced T1-weighted images. Minimum redundancy, maximum correlation, and least absolute shrinkage and selection operator were performed to select the features with the best performance in predicting preoperative CSF dissemination. A combined clinical-MRI radiomic prediction model was developed using multivariable logistic regression. Receiver operating curve analysis (ROC) was used to validate the predictive performance. Nomogram and decision curve analysis (DCA) were developed to evaluate the clinical utility of the combined model.

**Results:**

One clinical and nine radiomic features were selected for predicting preoperative CSF dissemination. The combined model incorporating clinical and radiomic features had the best predictive performance in the training cohort with an AUC of 0.89. This was validated in the internal and external cohorts with AUCs of 0.87 and 0.73. The clinical utility of the model was confirmed by a clinical-MRI radiomic nomogram and DCA.

**Conclusions:**

The combined model incorporating clinical, conventional MRI, and radiomic features could be applied to predict preoperative CSF dissemination for children with MB as a noninvasive biomarker, which could aid in risk evaluation.

**Supplementary Information:**

The online version contains supplementary material available at 10.1186/s12957-021-02239-w.

## Background

Medulloblastoma (MB) is the most common embryonal tumor located in the posterior cranial fossa. It usually affects young children before the age of 9 years [1]. Approximately 30% of children with MB have cerebral spinal fluid (CSF) dissemination with either magnetic resonance imaging (MRI) suggestive of disseminative nodules or CSF cytology demonstrated tumor cells [2].

At present, the standard treatment for MB is maximum resection followed by risk-adapted adjuvant chemoradiotherapy. Depending on the absence or presence of adverse prognostic factors including age younger than 3 years, anaplastic histopathologic subtype, CSF dissemination, and residual tumor greater than 1.5 cm in diameter, the child with MB will be stratified into an average or high-risk group [3]. The 2016 World Health Organization classification of tumors of the central nervous system (CNS) grades MB by its molecular profiling, which has a more reliable performance in stratifying the risk of MB and guiding clinical treatment strategies [4]. Although the advances regarding the molecular characteristics of MB could aid risk stratification, accurate identification of CSF dissemination remains important in prognosis prediction.

Both MRI of the CNS and CSF cytology will appear false positive and negative. On the one hand, MRI of the CNS is not able to detect early dissemination, while on the other hand, the equivocal findings such as enhanced meningeal thickening and nerve roots clumping may be misdiagnosed as dissemination [5, 6]. Because of technical or sampling problems, CSF cytology may fail to detect the tumor cells. Multiple CSF sampling may improve the diagnostic accuracy, but at the cost of additional discomfort to children as more lumbar punctures would be required [7]. Therefore, establishing a noninvasive biomarker for predicting CSF dissemination will be of great significance.

MRI is the modality of choice for risk stratification in children with MB. It can provide more information than conventional MRI features evaluated by radiologists, because images are not data mined [8]. To the best of our knowledge, there is no published data concerning the radiomics predicting the metastasis risk of MB in children.

Radiomics is an approach that is able to extract high-throughput quantitative features from medical images [9]. In theory, radiomic features are able to reflect biological characteristics of the tumor and could aide in differential diagnosis, and prognosis and distant metastasis prediction, among others [10–12]. In this present study, we investigated the added value of enhanced T1-weighted image-based radiomics to clinical characteristics in predicting preoperative CSF dissemination for children with MB.

## Materials and methods

### Patients

This retrospective study was approved by the Institutional Ethics Committee of Xinhua Hospital Affiliated to Shanghai Jiao Tong University School of Medicine (ethics approval number: XHEC-D-2020-136), and therefore, informed consent was waived.

The inclusion criteria included (i) the availability of preoperative head MRI with diagnostic quality, (ii) availability of spine MRI with diagnostic-quality performed pre- or postoperative but before adjuvant therapy, (iii) children without CSF dissemination confirmed by head and spine MRI required follow-up results for 1 year, and (iv) without any previous treatment. The exclusion criteria included (i) insufficient head and spine MRI quality, (ii) without 1-year follow-up results for children without CSF dissemination, (iii) previous treatment, and (iv) equivocal findings on spine MRI such as enhanced meningeal thickening and nerve roots clumping.

Children with pathologically confirmed MB were reviewed between November 2006 and November 2018. The children met the including and excluding criteria were randomly distributed to the training and internal validation cohorts according to a 7:3 ratio. A set of cases between December 2018 and February 2020 were used for external validation of the prediction model.

Clinical features, including age, gender, and histopathologic subtype (classic, desmoplastic-nodular, MB with extensive nodularity, and large cell/anaplastic) of all children with MB, were collected via medical records.

**Fig. 1:**
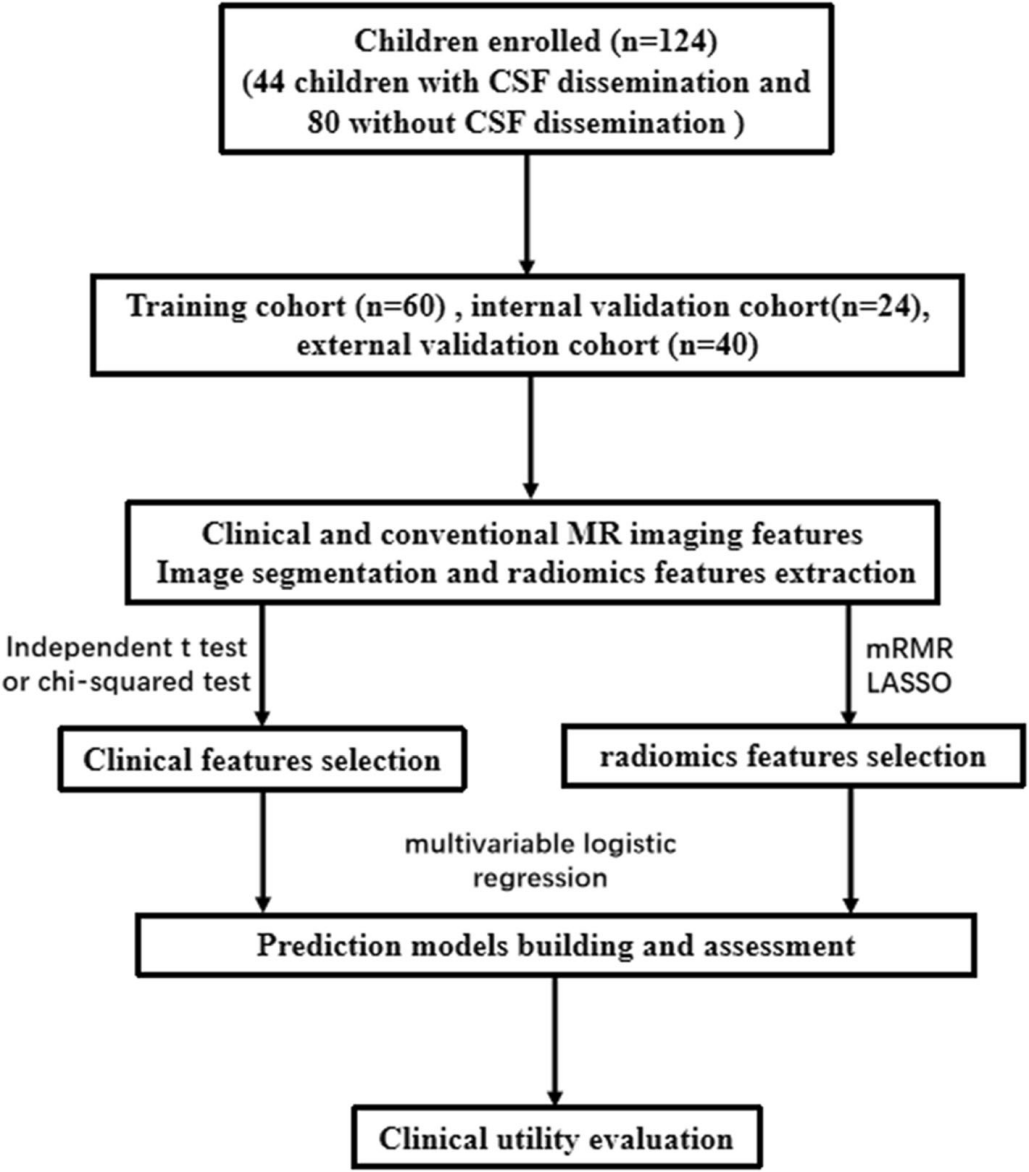
Workflow of this study. CSF cerebral spinal fluid; MRI magnetic resonance imaging; mRMR minimum redundancy and maximum correlation; LASSO least absolute shrinkage and selection operator

The workflow of this retrospective study is displayed in Fig. 1.

### Imaging acquisition

All of the children underwent pre- and postoperative contrast-enhanced T1-weighted head MRI using a 3.0-T MRI scanner (Signal HDxt, GE Healthcare, Boston, MA, USA) with an 8-channel head coil. The contrast-enhanced T1-weighted images were obtained with a slice thickness of 4 mm and a matrix of 512 × 512. Children also underwent contrast-enhanced spine MRI, which was given either preoperative or postoperative but before adjuvant therapy. Axial and sagittal-enhanced T1-weighted images were obtained with a thickness of 3 mm and gap of 0 mm. The detailed parameters for head and spine MRI are provided in Appendix E1 and E2.

The contrast material was administrated with a dose of 0.1 mmol/kg (Gadopentetate Dimeglumine, Beilu, Beijing, China). Children that were unable to remain motionless during the MRI examination were sedated with chloral hydrate (0.5 mg/kg).

### Qualitative image evaluation

#### Conventional MR imaging evaluation

All of the preoperative head MR images were reviewed by two pediatric radiologists (H.Z and J.N.L, with 9 and 12 years of experience, respectively, in pediatric neuroradiology). Discrepancies were resolved by consulting with a third pediatric neuroradiologist (Y.H.L) with 30 years of experience. A set of conventional MRI features were evaluated, including the location of the tumor, contrast enhancement pattern, intratumoral necrosis, hemorrhage, calcification, peritumoral edema, and the minimal apparent diffusion coefficient (minADC) value. A description for these features is provided in Appendix E3.

#### CSF dissemination assessment

The enhanced T1-weighted peri-surgical head and spine MR images were used for CSF dissemination assessment. Children with normal head and spine MR images and no subsequent dissemination at the 1-year follow-up were regarded as the non-CSF dissemination group. CSF dissemination was manifested as intracranial or intraspinal nodular lesions and leptomeningeal enhancement. Children with equivocal findings on head and spine MR images were excluded.

### Image segmentation and radiomic feature extraction

**Fig. 2:**
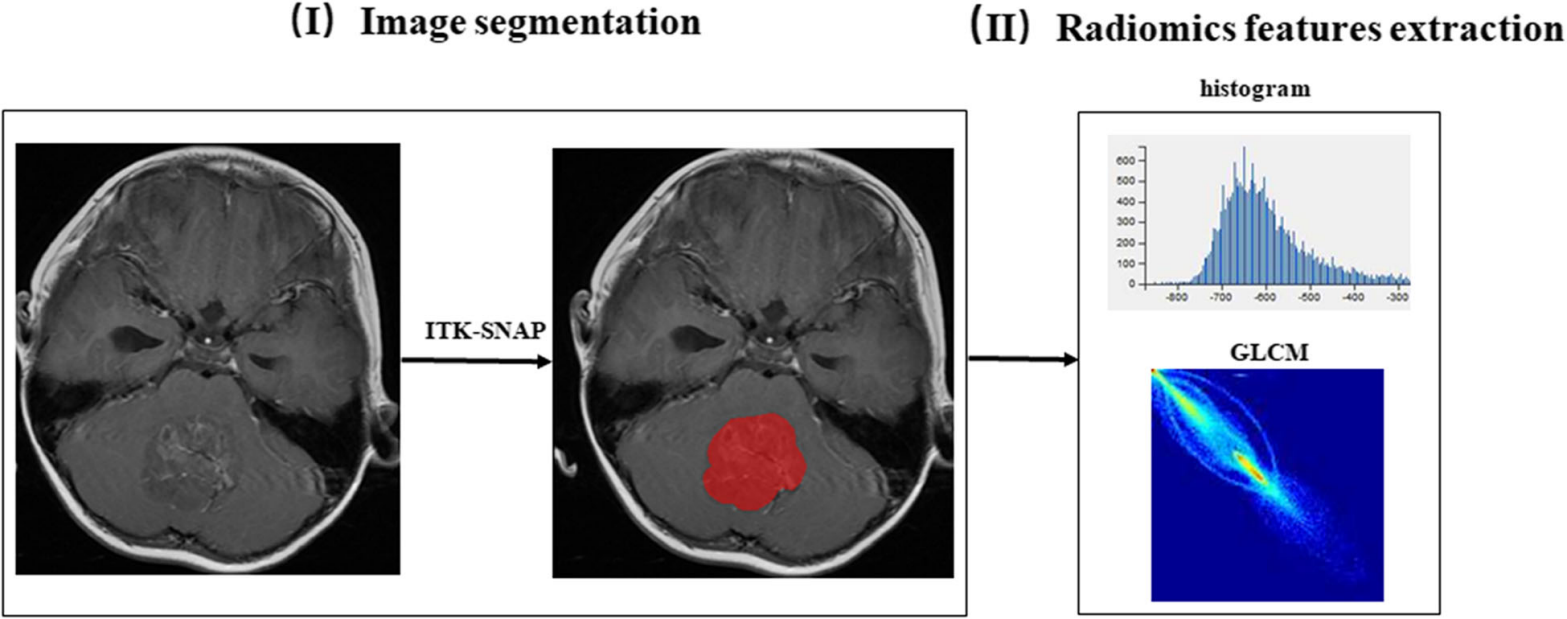
Workflow of image segmentation and radiomic feature extraction. (I) The left panel shows representative tumor slices. The region of interest was delineated manually slice by slice and then a 3D VOI was generated as shown in the image on the right. (II) Radiomic features were extracted from the VOI including histogram parameters, volume and shape parameters, and texture features

The delineation of MB and radiomic extraction is shown in Fig. 2. One radiologist (H.Z with 9 years of experience in pediatric neuroradiology) determined the volume of interest (VOI) of the tumor on preoperative contrast-enhanced T1-weighted images using software package ITK-SNAP (www.itksnap.org). The peritumoral edema and surrounding vessels were carefully avoided. The segmentations were examined by another pediatric neuroradiologist (M.L with 20 years of experience). Both were blinded to the CSF dissemination status.

By using an in-house software Analysis Kit (GE healthcare), a total of 385 radiomic features were extracted automatically from the VOI including histogram parameters, volume and shape parameters, Haralick features, gray-level co-occurrence parameters, and gray-level run-length matrix parameters.

### Feature selection, prediction model building, and validation

Differences in clinical and conventional MR imaging characteristics between the children with and without CSF dissemination in the training, internal, and external validation cohorts were assessed. A clinical prediction model was built using the features with significant differences.

In the training cohort, minimum redundancy and maximum correlation (mRMR) and least absolute shrinkage and selection operator (LASSO) were performed to reduce dimension and select the radiomic features with the strongest CSF dissemination-related correlation. The radiomic score (rad-score) of each child with MB was calculated by adding all of the products of the selected features and their corresponding coefficients, after which a radiomic prediction model was built. A combined clinical-radiomic prediction model was developed using multivariable logistic regression, which incorporated selected clinical features and rad-scores. A clinical-radiomic nomogram was then constructed in the training cohort.

The receiver operating characteristic (ROC) curves were used to evaluate the predictive performance of the clinical, radiomic, and combined models in both the training and two validation cohorts. The area under the curves (AUC), sensitivities, specificities, and accuracy were calculated. And then, the Hosmer-Lemeshow test was performed to assess the goodness-of-fit of the combined model. Decision curve analysis (DCA) was implemented to determine the clinical usefulness of the prediction models in the training cohort at different threshold probability.

### Statistical analysis

The differences of clinical and conventional MRI characteristics between the training and two validation cohorts, as well as between children with and without CSF dissemination in their respective cohorts were evaluated using independent *t* tests or chi-squared tests according to the type of the data. The optimal values of the ROCs were determined using Youden’s index. Delong’s test was used to assess the differences in the AUC values between the clinical and clinical-radiomic combined models.

All statistical analyses were performed using the R software package (version 3.4.2, http://www.Rproject.org). The ROC curves were performed using the “pROC” package. Multivariate logistic regression was plotted with the “rms” package. The Hosmer-Lemeshow test was conducted using the “Resource Selection” package. DCA was developed with the function of “dca.R”. *P* < 0.05 was set as statistical significance.

## Results

### Clinical features of the children

According to the inclusion criteria, a total of 124 children with MB were recruited in this study (60 children in the training cohort, 24 and 40 children in the internal and external validation cohorts, respectively). None of the children experience extra-CNS metastases. Forty-four children (35.6%) were confirmed to have CSF dissemination by head and spine MRI evaluation. Five children had only intracranial dissemination while 39 children had both intracranial and intraspinal dissemination. Eighty children without CSF dissemination were identified by MRI evaluation and follow-up information.

The clinical features of the children in the training and two validation cohorts were displayed in Table 1. There were no significant differences among the three cohorts in terms of clinical features except for histopathological subtype. In the training cohort, children were significantly younger (*P*=0.028) in the CSF dissemination group than in the non-CSF dissemination group.

**Table 1:** Clinical and conventional MRI characteristics of children with MB

Characteristics	Training cohort (n = 60)		*p* value	Internal validation cohort (*n* = 24)		*p* value	External validation cohort (n = 40)		*p* value
	Non-metastasis (*n* = 38, %)	Metastasis (*n* = 22, %)		Non-metastasis (*n* = 15, %)	Metastasis (*n*= 9, %)		Non-metastasis (*n* =27, %)	Metastasis (*n*= 13, %)	
Age (mean ± SD, years)	6.2 ± 3.5	4.3 ± 2.5-	0.028	5.1 ±2.9	3.6 ±1.7	0.154	6.3 ±3.4	6.4 ±3.3	0.937
Gender (%)			1.000			0.597			0.360
Male	26 (68.4)	15 (68.2)		7 (46.7)	6 (66.7)		13 (48.1)	9 (69.2)	
Female	12 (31.6)	7 (31.8)		8 (53.3)	3 (33.3)		14 (51.9)	4 (30.8)	
Histopathologic subtype (%)			0.632			0.444			0.748
Classic	33 (86.8)	17 (77.3)		10 (66.7)	8 (88.9)		21 (77.8)	11 (84.6)	
Large cell/anaplastic	1 (2.6)	1 (4.5)		4 (26.7)	1 (11.1)		1 (3.7)	0 (0)	
Desmoplastic-nodule	4 (10.5)	4 (18.2)		1 (6.7)	0 (0.0)		5 (18.5)	2 (15.4)	
Location (%)			1.0000			0.507			1
Midline	34 (89.5)	20 (90.9)		15 (100.0)	9 (100.0)		24 (88.9)	11 (84.6)	
Nonmidline	4 (10.5)	2 (9.1)		0 (0.0)	0 (0.0)		3 (11.1)	2 (15.4)	
Enhancement pattern (%)			0.4032			0.052			0.399
Diffuse	23 (60.5)	17 (77.3)		8 (53.3)	9 (100.0)		17 (63.0)	10 (76.9)	
Incomplete	10 (26.3)	3 (13.6)		4 (26.7)	0 (0.0)		3 (11.1)	2 (15.4)	
Minimal	5 (13.2)	2 (9.1)		3 (20.0)	0 (0.0)		7 (25.9)	1 (7.7)	
Necrosis or cyst(%)			0.8664			0.729			0.311
None	5 (13.2)	2 (9.1)		1 (6.7)	0 (0.0)		0 (0)	1 (7.7)	
Small	22 (57.9)	14 (63.6)		8 (53.3)	5 (55.6)		22 (81.5)	9 (69.2)	
Both	11 (28.9)	6 (27.3)		6 (40.0)	4 (44.4)		5 (18.5)	3 (23.1)	
minADC (mean ± SD) mm^2^/s)	0.4 ± 0.1	0.4 ± 0.1	0.191	0.5 ± 0.1	0.4 ± 0.1	0.0212	0.5 ± 0.1	0.4 ± 0.1	0.171

*minADC* minimal apparent diffusion coefficient

### Feature selection, prediction model building, and validation

**Fig. 3:**
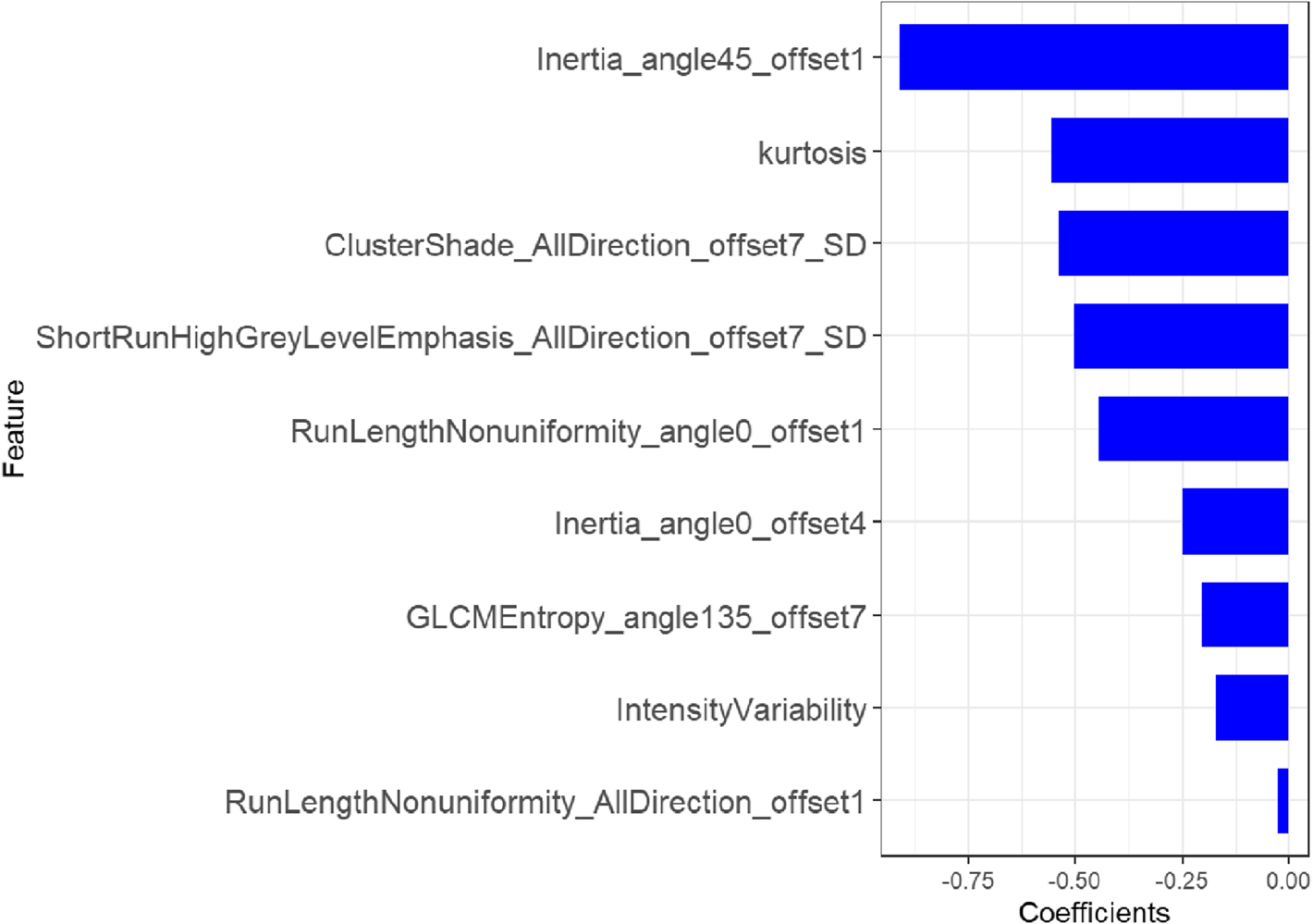
The selected radiomic features. The role of selected features contributing to the developed rad-score is shown. The selected features are plotted on the *y*-axis, and their regression coefficients in the LASSO analysis are plotted on the *x*-axis

In the training cohort, nine radiomic features with the strongest CSF dissemination features were selected using mRMR and LASSO shown in Fig. 3. The rad-score was calculated using the following formula:

Rad − score =  − 0.557 × Kurtosis

−0.502 × ShortRunHighGreyLevelEmphasis _ AllDirection _ offset7 _ SD

−0.538 × ClusterShade _ AllDirection _ offset7 _ SD

−0.444 × RunLengthNonuniformity _ angle0 _ offset1

−0.909 × Inertia _ angle45 _ offset1

−0.248 × Inertia _ angle0 _ offset4

−0.026 × RunLengthNonuniformity _ AllDirection _ offset1

−0.204 × GLCMEntropy _ angle135 _ offset7

−0.17 × Intensity/Variability − 0.735

**Fig. 4:**
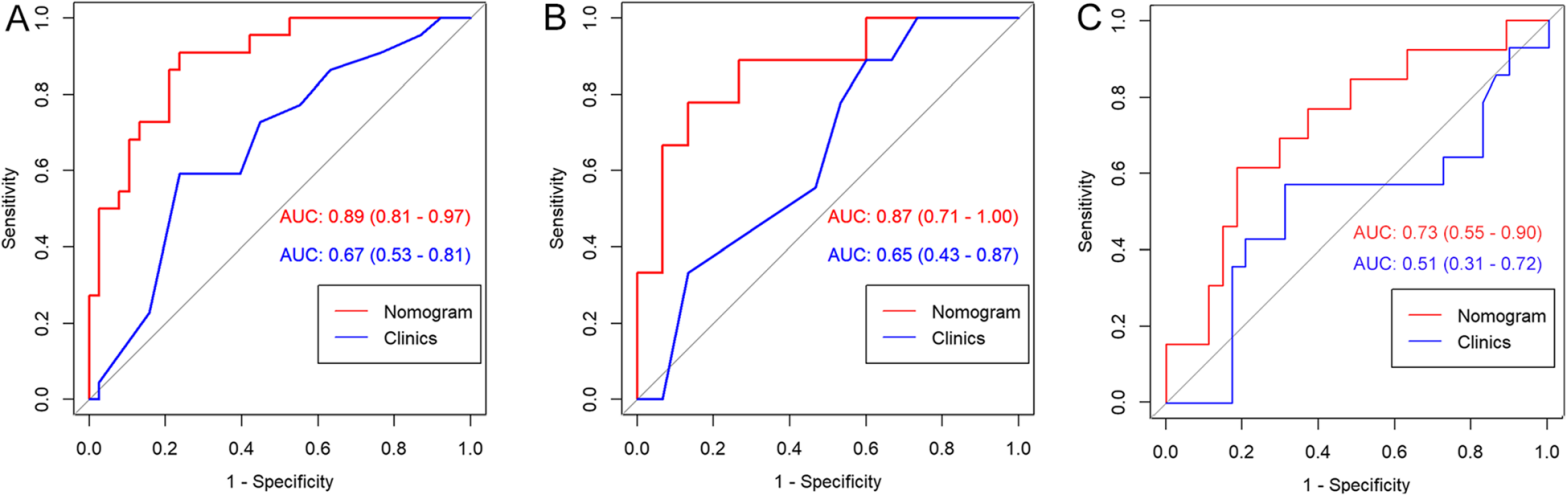
ROC curves of the traditional feature model and nomogram. **a** The AUC was significantly different (0.67 and 0.89, respectively) between the clinical model and the nomogram in the training cohort. **b**, **c** The AUC of the nomogram was significantly higher than a clinical model in the internal and external cohorts. Delong’s test showed that the differences between the ROC curves of the nomogram and clinical model were significantly different in both the training and two validation cohorts. ROC receiver operating characteristic, AUC area under the curve

**Fig. 5:**
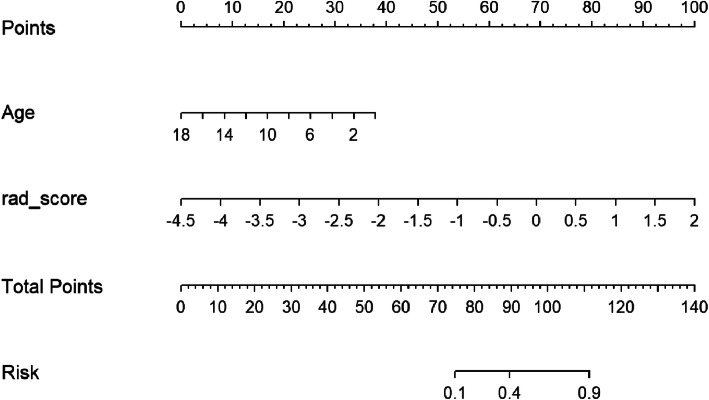
The developed clinical-radiomic nomogram for predicting CSF dissemination

The combined clinical-radiomic predictive model was developed using multivariate logistic regression analysis. The AUC of the value of the combined model was 0.89. And the AUC of the clinical model was much lower (0.67), which indicated that radiomic features could improve performance. The results of the combined predictive model in the training and two validation cohorts were shown in Table 2 (Fig. 4). The nomogram was built with age and rad-score in the training cohort (Fig. 5).

**Table 2:** Performance of the combined clinical-radiomic predictive model

	Training cohort	Internal validation CohortcohorCohort	External validation cohort
AUC (95%)	0.89 (0.81–0.97)	0.87 (0.71–1.00)	0.73 (0.56–0.85)
Accuracy	0.82	0.83	0.73
Sensitivity	0.91	0.87	0.78
Specificity	0.76	0.87	0.85

**Fig. 6:**
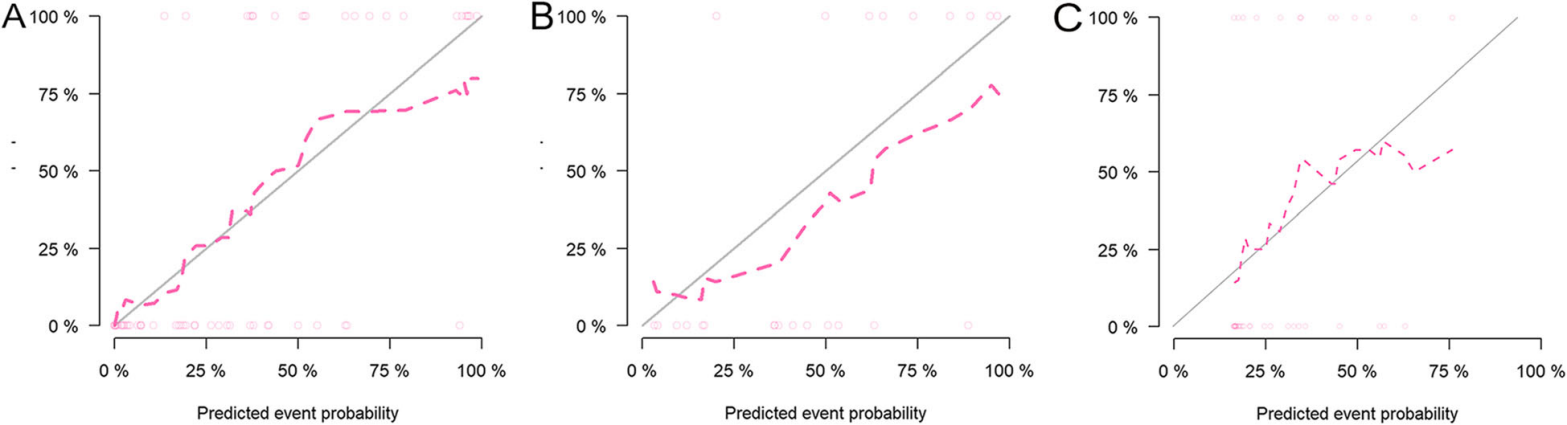
Results of the Hosmer-Lemeshow test. The combined model fit well with the real situation both in the training cohort (**a**), internal and external validation cohorts (**b**, **c**)

**Fig. 7:**
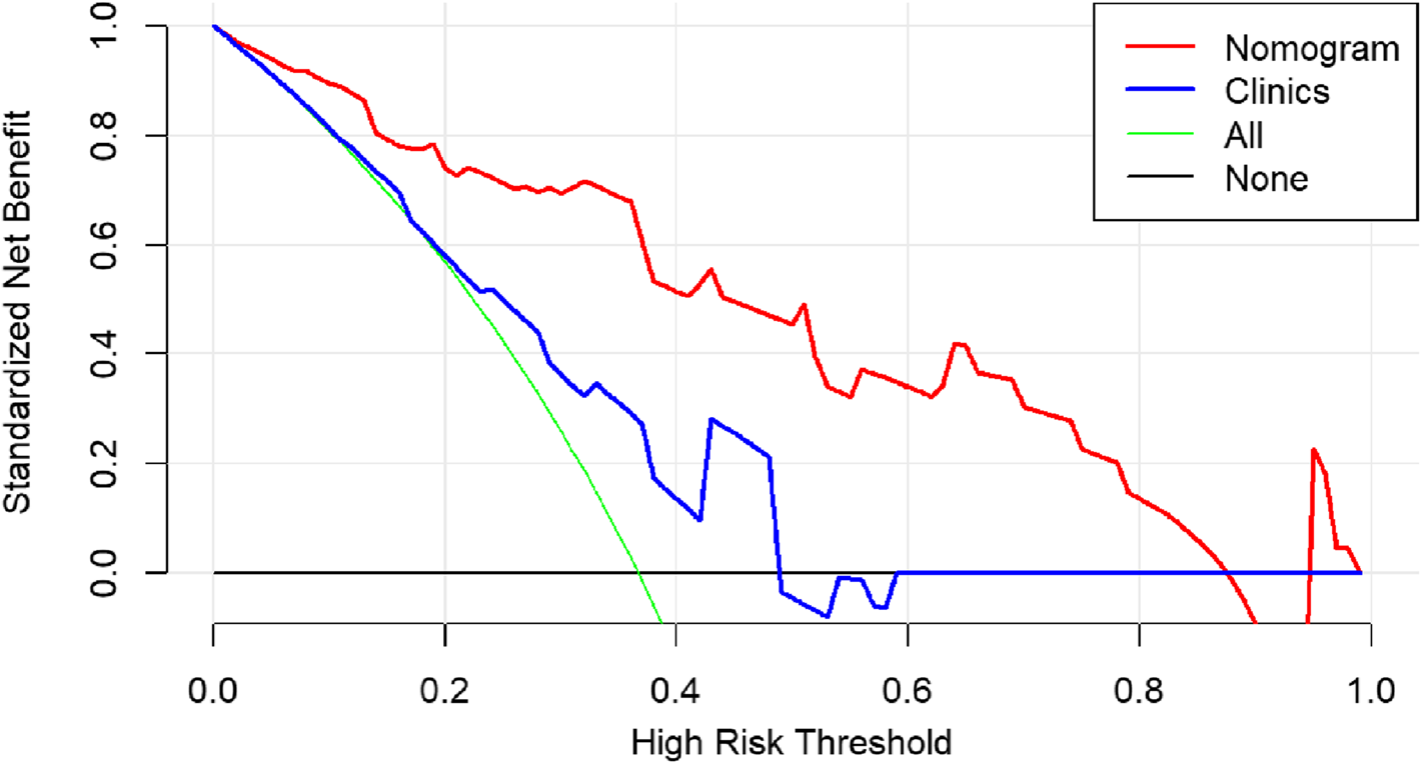
Decision curve analysis demonstrated that the combined model had a higher net benefit than the traditional model at every threshold probability

The CSF dissemination predictive performance of combined prediction model was also robust when applied to the internal and external validation cohorts with an AUC of 0.87 and 0.73, respectively. The Hosmer-Lemeshow test demonstrated that the goodness-of-fit of the combined model was high in both the training and two validation cohorts (Fig. 6). DCA revealed that at every threshold probability, it was more beneficial using the combined model than the clinical model alone (Fig. 7).

## Discussion

In the present study with a relatively large case group (*n*=124), we developed and validated a clinical-radiomic combined model for preoperative prediction of CSF dissemination in children with MB. The nomogram combined the age of the children and a preoperative-enhanced T1-weighted image-based radiomic signature. The results demonstrated that compared with the prediction model built using clinical features alone, the radiomic signature could improve the predictive performance of CSF dissemination. The AUC was increased from 0.67 to 0.89 in the training group. The sensitivity, specificity, NPV, and PPV were relatively higher both in the training and two validation cohorts. The higher sensitivity indicated that the combined model could identify children with CSF dissemination accurately. These children should undergo examinations with a higher specificity, such as CNS, MRI, and CSF cytology to evaluate the dissemination staging.

Children with CNS dissemination should be given aggressive therapy, as prognosis is usually poor, no matter what the molecular subgroup is. It is, therefore, paramount to establish a method to accurately diagnose CNS dissemination. Traditionally, diagnosis of CSF dissemination encompassed comprehensively analyzing the combined results of CNS, MRI, and CSF cytology [7, 13], both of which may over- or underestimate the CSF dissemination. On the one hand, overestimation leads to higher risk stratification and unnecessarily high radiotherapy doses, which could affect the quality of life of children with MB. On the other hand, underestimation leads to lower risk stratification and lower radiotherapy doses, which could increase the possibility of tumor recurrence and decrease survival. Identification of tumor cells by CSF cytology is an important element of the diagnosis of CSF dissemination. However, a negative result must be viewed with caution due to high rates of technique and sampling errors [14]. Multiple lumber punctures may improve tumor cell detection, but cause increased pain to children and also delays corresponding adjuvant treatment.

In this retrospective study, CSF cytology results were not obtained. Therefore, the diagnosis of CSF dissemination depended on head and spine MR images. Children without CSF dissemination were identified by negative head and spine MRI and outcome at the 1-year follow-up. This diagnosis strategy is consistent with the literature determining the status of CSF dissemination by using overall survival [6]. The equivocal findings in spine MRI such as linear enhancement, clumping, and enhancement of nerve roots can be misdiagnosed as CSF dissemination, all of which can be identified in preoperative MRI. Therefore, extreme care is needed and close follow-up is required to confirm any initial diagnosis.

Radiomics can evaluate the intratumoral heterogeneity and predict prognosis by extracting high throughput quantified features using medical images. A previous study indicated that when the prediction accuracy of the radiomic signature was constructed by using 24 radiomic features from preoperative computerized tomography images for lymph node metastasis in patients with colorectal cancer, the concordance index was 0.773 [15]. In another study, when the radiomic signature was constructed using T2-weighted images, the prediction accuracy was improved compared with a model built only using clinical features for synchronous distant metastasis in patients with rectal cancer, where the AUC for the combined model and clinical model was 0.827 and 0.779, respectively [12].

In CNS tumors, radiomics is mainly applied to differential diagnosis of tumors, World Health Organization grading, molecular profiling, and prognosis prediction in the adult population [16–19]. However, for pediatric MB, research is focused on differential diagnosis of tumors in the posterior cranial fossa and in molecular subtype prediction [20, 21]. Therefore, it is worth exploring an imaging biomarker to detect CSF dissemination based on the MRI radiomic features of pediatric MB.

According to previous reports, the CSF dissemination probability of medulloblastoma has a close relationship to molecular subgroups. For example, the prognosis of children with group 3 is very poor and prone to early metastasis [22]. Some MRI features of MB are reliable to predict molecular subgroups, especially their location and degree of enhancement. Group 3 tumors are mainly located in the midline of posterior cranial fossa with strong enhancement, whereas group 4 tumors, although also located at the midline, show minimal enhancement [23]. These imaging features are able to be assessed on enhanced T1-weighted images, and as such, we chose to use this type of enhancement to extract radiomic features.

In the present study, the results demonstrated the added value of radiomics to clinical and conventional MRI characteristics in predicting CSF dissemination for pediatric MB. The higher sensitivity and accuracy of the combined model compared with the clinical model alone is encouraging. It is likely that the probability of CSF dissemination could be calculated using the proposed combined model nomogram.

The CSF dissemination prediction model included the clinical variable of age, as group 3 tumors are prone to occur in younger children and infants [24]. Regarding to radiomic features, most of the selected features used to determine the rad-score were texture features. This is consistent with the better performance of texture features compared with first-order features in tumor prognosis prediction, as these features may reflect intratumoral heterogeneity [25].

There are several limitations to the current study. First, due to the small sample size of the cohort, useful conventional MRI features for the predictive model could not be used because of selective bias. The association between conventional MRI features and CSF dissemination in children with MB should be further explored in a large-scale study. Second, due to the retrospective nature of the study, an external validation from another institution was not performed. A multi-center prospective research with different MRI scanning protocols is needed to evaluate the generalization of our predictive model. Third, only enhanced T1-weighted images were used in this radiomic research. Multiparametric MRI including T2-weighted images should be implemented into the predictive model to improve its robustness. Finally, we were not able to obtain CSF samples in the majority of their patients to identify M1 disease. But because of technical or sampling problems, CSF cytology may fail to detect the tumor cells. A more precise technique called circulating tumor DNA should be used in further study to improve the performance of the predictive model.

## Conclusions

In summary, our preliminary study demonstrated that a preoperative-enhanced T1-weighted image-based radiomics could improve the prediction accuracy of CSF dissemination in children with MB. A nomogram constructed based on the radiomic signature and selected clinical features may provide more useful information for clinical decision making.

## Supplementary Information

**Additional file 1..**

## Data Availability

The datasets generated and/or analyzed during the current study are not publicly available due to hospital policy but are available from the corresponding author on reasonable request.

## Abbreviations

MB: Medulloblastoma
CSF: Cerebral spinal fluid
