# Supplementary material for: Clinical-MRI radiomics enables the prediction of preoperative cerebral spinal fluid dissemination in children with medulloblastoma
Source: World J Surg Oncol. 2021 Apr 22;19:134. doi: 10.1186/s12957-021-02239-w (PMC8063474; doi:10.1186/s12957-021-02239-w)
Supplement: Supplementary file 1 — Additional file 1.. [file 12957_2021_2239_MOESM1_ESM.docx]

**Appendix E1** Main parameters for head MRI

(1) Diffusion-weighted imaging (DWI): Repetition time (TR) = 2752 ms, echo time (TE) = 98.3 ms, inversion time (TI) 2752 ms, slice thickness = 5 mm, b value = 0,1000 s/mm^2^; (2) axial T1 FLAIR: TR = 1800 ms, TE = 20.0 ms, TI = 800 ms, slice thickness = 4 mm, increment = 0 mm；(3) axial T2 FLAIR: TR = 7000 ms，TE = 120 ms, TI = 2250 ms, slice thickness=4 mm, increment = 0 mm; (4) sagittal T2 weighted images: TR =2500 ms, TE = 80 ms, slice thickness = 4 mm, increment = 0 mm. (5) The parameters for contrast enhanced axial, coronal and sagittal T1 weighted images were the same as the nonenhanced T1 weighted images.

**Appendix E2** Main parameters for spine MRI

(1) Sagittal T2 weighted images: TR = 2540 ms, TE = 116 ms, slice thickness = 3 mm, increment = 0.5 mm; (2) sagittal T1 weighted images: TR = 600 ms, TE = 11 ms, slice thickness = 3 mm, increment = 0.5 mm; (3) contrast enhanced sagittal T1 weighted images: TR 740 ms, TE = 11 ms, slice thickness = 3 mm, increment = 0.5 mm; and (4) contrast enhanced axial T1 weighted images: TR = 660 ms, TE =11 ms, slice thickness = 3 mm, increment = 0.5 mm.

**Appendix E3** Conventional MRI feature characterization

The location of the tumor was defined by its epicenter in relation to the midline of the posterior fossa. The degree of contrast enhancement was evaluated by the proportion of the solid portion showing uptake of contrast media (minimal enhancement: ≤ 1/3, incomplete: 1/3–2/3, diffuse: ≥ 2/3). The presence of hemorrhage was evaluated by high intensity on T1 weighted images and low intensity on T2 weighted images, especially if the presence of fluid-fluid level in the tumor and blooming on susceptibility weighted imaging (SWI) was found. Tumor calcification was either focal or irregular high density on computed tomography scan and was low intensity on T2 weighted images, which a tumor size akin to that found on SWI. The extent of the peritumoral edema was defined by the widest edema zone (non or minimal < 1.5 cm, obvious ≥ 1.5 cm). The non-enhancing portion, with high intensity on T2 weight images, was either the necrotic or cystic region. The tumor was considered as large if the area was more than 1 cm^2^. The lowest apparent diffusion coefficient (ADC) of all of the ROIs was defied as minADC.
